# Quantitative Measurement of the Tack for Carbon Fiber Reinforced Epoxy Prepreg by Using a Compression-to-Tension Method

**DOI:** 10.3390/ma18215050

**Published:** 2025-11-06

**Authors:** Xueming Wang, Guoli Li, Xiu Liu, Xiaofeng Lin, Baolin Pang

**Affiliations:** 1 Composite Test and Technology Center, AVIC Composite Corporation Ltd., Beijing 101300, China; wtj1998@163.com (X.W.);; 2 AVIC Composite Technology Center, AVIC Manufacturing Technology Institute, Beijing 101300, China

**Keywords:** carbon fiber, composites, prepreg tack, fabric prepreg, unidirectional prepreg

## Abstract

Prepreg tack is an important process quality parameter for prepregs during laying. Aiming at the current lack of standardized testing for prepreg tack, this paper established a quantitative testing method for prepreg tack—a compression-to-tension method—and proposed a parameter of Compression Tack Index as a quantitative evaluation index for prepreg tack. The prepreg/prepreg tack and prepreg/metal tack of carbon fiber reinforced epoxy prepregs were evaluated and the applicability of this compression-to-tension method was verified, comparing it with the qualitative testing method by vertical metal plates. The results show that the compression-to-tension method is suitable for quantitative testing of the tack for unidirectional prepregs and fabric prepregs, with good repeatability and stability of test results, and is not affected by personnel changes. Considering that tack characterization based only on the separation process cannot accurately evaluate the tack of different materials, Compression Tack Index is an accurate parameter that characterizes the prepreg tack because it can reflect the process of tack formation and tack separation. Compared with the vertical metal plate method, the discrimination of the test results by the compression-to-tension method is significant. The tack of the slitting prepreg without polyethylene film coating is lower than that of the mother prepreg (one-meter-width prepreg) with polyethylene film.

## 1. Introduction

The manufacturing technology of advanced carbon fiber reinforced resin-based composites is a key technology for achieving lightweight aircraft structures. The carbon fiber prepreg is laid on the mold mainly through manual or automatic placement technology, including Automated Fiber Placement (AFP) and Automated Tape Laying (ATL), and then cured in the autoclave [1]. In order to keep the prepreg in the desired position on the mold, the prepreg should have an appropriate tack and a better drapability or deformability. The appropriate tack of the prepreg is an important guarantee of the successful placement of the prepreg on the surface of the mold. It has a significant influence on the macroscopic and microscopic stacking defects of composites and their mechanical properties after curing. High temperature resistance and high toughness are the two main development directions of advanced carbon fiber reinforced resin-based composites. However, the increase in toughness leads to a decrease in the process performance, which affects the laying efficiency and product quality. The prepreg for automatic laying should meet the requirements of excellent process performance and good comprehensive mechanical properties. Therefore, in order to ensure the laying process of prepregs, Boeing Material Specification (BMS8-276N) is willing to sacrifice mechanical properties in exchange for good material process performance, such as adjusting the residual compressive strength after impact (CAI) of composites from 310 MPa to 280 MPa for manual and Automatic Tape Laying (ATL) processes, and 250 MPa for Automated Fiber Placement (AFP) [2]. Therefore, the prepreg tack has become an important process quality indicator for prepregs, especially in AFP manufacture-induced defect generation.

Usually, manufacturers of structural materials provide very little information on the tack properties of their commercially distributed prepregs. The prepreg tack is usually limited to “low”, “medium”, and “high” tack or tack level grading [3]. The tack grading method cannot accurately characterize the prepreg tack because this method is greatly affected by environmental and personnel factors. During engineering applications, the relationship between the process parameters and the prepreg tack depends on the experience of the process personnel. Therefore, it is particularly important to propose a widely accepted testing method to quantitatively test and evaluate the prepreg tack. Currently, there are many studies on a single quantitative testing method for the prepreg tack aimed at analyzing and validating AFP and ATL equipment and processes. For example, based on the principle of pressure-sensitive adhesive (PSA) testing, various quantitative testing methods have been established, including a probe test method [4], a floating roller stripping method according to an ASTM D3167 method [5], a continuous application-and-peel test method [6], and a tension-shear test method [7]. Dubois [8] designed a probe tack test apparatus to characterize the tack of carbon-epoxy prepregs using the maximum debonding force as a characterization. Desire [9] applied the probe method to establish an empirical correlation between the tack of phenolic prepregs and the rheological state of the base resin. Most of the studies reported in the early studies [10,11,12] applied the probe test method to examine the tack between the prepreg and the metal. A modified probe test method which is capable of inter-ply and ply-tool tack measurement was proposed by Wang [13]. To characterize the material for automated material layup, the continuous application-and-peel test method first proposed by Crossley [14,15] has been developed into the ASTM D8336-24 [16], measuring the tack specified in terms of a peel force at a given specimen width. The repeatability and reproducibility of the results of the test method [17], and the development of ASTM D8336-21 [18], have been reported in recent years.

Due to the presence of fibers and the viscoelastic property of prepregs, there is a significant difference between the prepreg tack test and the pressure-sensitive adhesives test. It requires comprehensive consideration of various influencing factors, and due to limitations in testing conditions and research objectives, various testing methods have certain limitations. The three methods mentioned above each have their own shortcomings. For the probe test method, tack measurement is sensitive to the probe surface and is sensitive to surface roughness changes caused by fiber morphology and resin impregnation distribution in large samples [8]. For a continuous application-and-peel test method, such as ASTM D8336-24, it is difficult to obtain the intrinsic prepreg tack because the average peeling force includes not only the debonding force of the prepreg itself, but also the bending deformation force of the prepreg. The fiber stiffness limits the experimental range. If the peel angle is large, the interface between the prepreg and the support can instantly fail without peeling, and high-tack prepreg is prone to roller detachment [19]. For the tension-shear test method, the stress distribution of the test sample is relatively complex, which can easily cause stress concentration and cannot truly reflect the prepreg tack. It is noted that most of the studies reported in the literature using the tack test methods apply the maximum debonding force to characterize the prepreg tack. The maximum debonding force only reflects the tack characteristics of the prepreg during the separation process. In fact, the prepreg tack is closely related to the tack formation process. Therefore, it is necessary to establish a new testing method and propose a physical parameter that can reflect the tack formation process and the tack separation process of the prepreg.

This paper establishes a compression-to-tension method to quantitatively test the prepreg tack, compares it with the qualitative testing method for vertical metal plates by testing the tack of carbon fiber reinforced epoxy prepregs, and verifies the repeatability, stability, and applicability of the testing method. The research results provide important guidance and have reference significance for the process quality evaluation of prepreg tack.

## 2. Experimental Details

### 2.1. Material and Equipment

In this paper, three types of carbon fiber reinforced epoxy prepregs are applied: two types of unidirectional prepregs named MX and AX and one fabric prepreg named MX. The three types of prepreg, supplied by AVIC Composite Corporation Ltd. (Beijing, China), are made from epoxy resins with T800-grade carbon fibers. The fabric type is 2 × 2 twill structure. The main components of MX resin are a mixture of bisphenol A epoxy and tetrafunctional epoxy. AX resin is bisphenol A epoxy resin. The parameters of these materials are listed in Table 1. The equipments applied in this paper include a universal testing machine INSTRON 3342 (Norwood, MA, USA) with an accuracy of ±0.5%, a scanning electron microscope (SEM, Quattro S, Hillsboro, OR, USA) and a Micro CT (nano Voxel2000, Tianjin, China).

CT testing process: 4 layers of 50 mm × 50 mm prepreg sample were applied, compressed under the compression conditions (Voltage 80 kV; Current 20 uA; exposure time 0.45 s; resolution 25 μm) described in this paper, and applied to the sample stage. At the same time, the sample was placed in the CT radiation chamber, and dynamically scanned. The center position of the sample was adjusted, and then rotated 1080 degrees to scan. We collected 2D images and saved them, exported the data, and used data reconstruction software for 3D modeling.

### 2.2. Tack Measurement

#### 2.2.1. Conditioning and Preparation of Samples

Test environment: temperature (23 ± 2) °C, relative humidity (50 ± 10)%.

After the prepreg is released from the refrigerated state, it should be left at room temperature for at least 6 h. After it returns to room temperature, it should be checked that there is no condensed water vapor outside the packaging before opening the packaging bag and taking a sample as soon as possible.

The sample should be cut at least 25 mm away from the edge of the prepreg. For unidirectional prepregs, the sample length should be parallel to the fiber direction; for fabric prepregs, the sample length should be cut at a 45° angle to the fiber direction. The sample should retain a double-sided covering of isolation polyethylene (PE) film (or release paper), and should completely cover the prepreg. The sample should be flat, without bubbles, wrinkles, or raised corners.

#### 2.2.2. Compression-to-Tension Method

**Figure 1:**
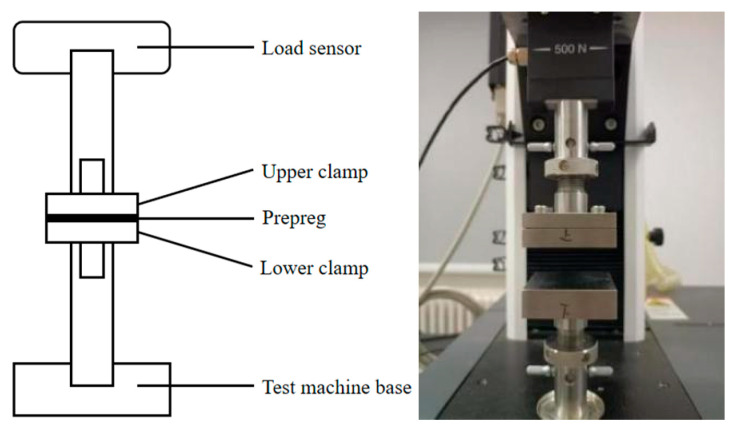
Physical device and schematic diagram of the compression-to-tension test method.

Prepreg samples with a size of (50 ± 0.5) mm × (50 ± 0.5) mm were pasted onto the surface of the fixture with double-sided adhesive tape. The tack between prepreg and prepreg is expressed by prepreg/prepreg tack, and the tack between prepreg and other material is expressed by prepreg/other material (such as prepreg/metal). For the prepreg/prepreg tack test, two prepreg samples were pasted on the upper and lower surfaces of the fixture, respectively. For the prepreg/metal tack test, only one prepreg sample was pasted on the lower surface of the fixture. The testing fixture consisted of two square stainless steel plates, with a specification size of (50 ± 0.5) mm × (50 ± 0.5) mm. The test fixture was installed on the universal testing machine INSTRON 3342 (Norwood, MA, USA). The up and down movement of the fixture was controlled by the universal testing machine to conduct a compression-to-tension test. The experimental device and schematic diagram are shown in Figure 1.

**Figure 2:**
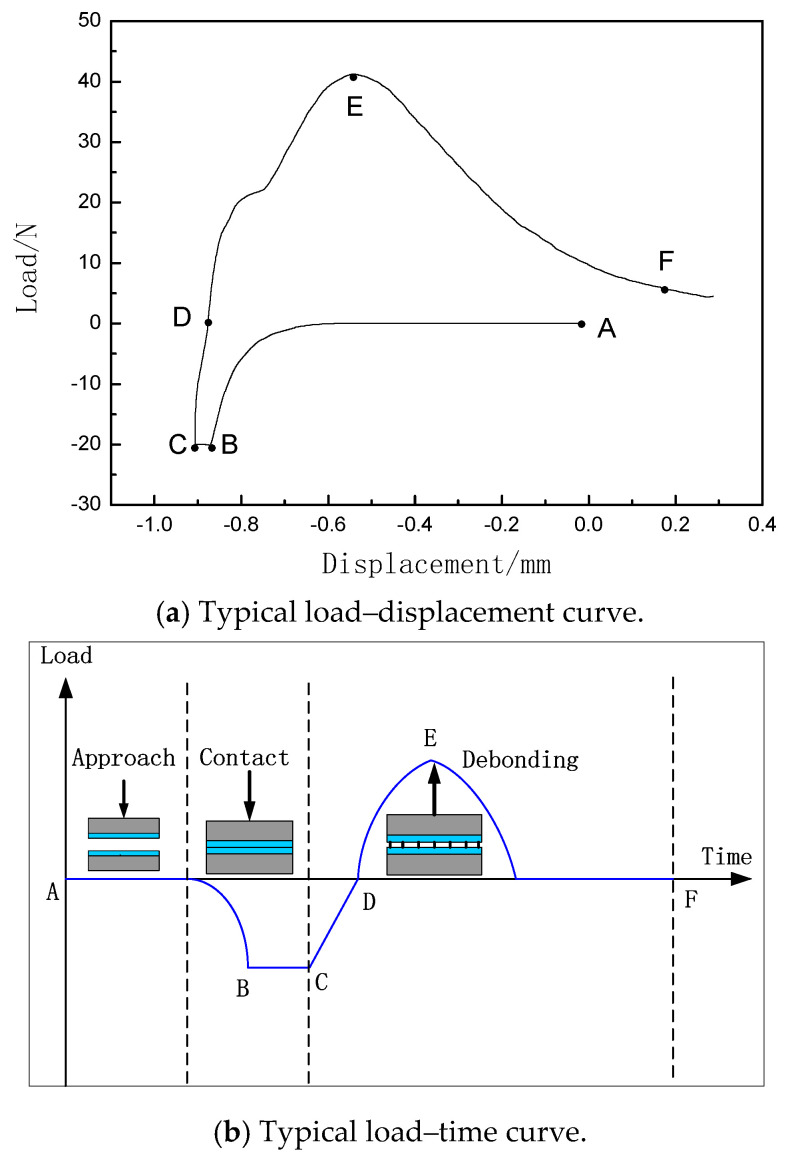
Load–displacement curve and load–time curve during compression-to-tension testing process.

According to the stress state of the prepreg, the entire experimental process can be divided into three periods: compression (loading), holding, and tension (unloading). In a standard test cycle, the compression rate is set to 1 mm/min in the compression period, and the prepreg sample is compacted to a pressure of 20N controlled through testing machine program settings, keeping the pressure constant for a duration of 30 s during the holding period. During the tension period, the tensile rate is 3 mm/min. The typical load–displacement and load–time curves are recorded to study the changes in prepreg tack, as shown in Figure 2. All average measured loads for each test were obtained from five prepreg specimens.

During the holding period (BC stage), the prepreg stack undergoes compression, and energy is absorbed into the prepreg stack. In the tension period (CE stage), the prepreg stack is subjected to a tensile load, which continues to increase at a constant rate through the zero-stress point (“D” position), and the energy absorbed in the compression/holding period begins to be released. The prepreg tack is influenced by the whole process, which is jointly determined by the material and the experimental operation characteristics. The prepreg tack measurement can be regarded as a separation energy, which is influenced by materials and test parameters.

For the quantitative evaluation of prepreg tack under different materials and test parameters, this paper proposes the parameter of Compression Tack Index (*CTI*), which describes the ratio of the output energy (i.e., tensile energy) of the prepreg stack during tensile unloading to the input energy (i.e., compressive energy) of the prepreg stack during compressive loading. It can be expressed by Equation (1):

(1) $$CTI=W_{adh}/W_{pre}$$

where *W_adh_* is the tensile energy and *W_pre_* is the compressive energy. *W_adh_* and *W_pre_* can be expressed by Equation (2) and Equation (3), respectively:

(2) $$W_{adh}=\int_{D}^{F}f(x)dx$$

(3) $$W_{pre}=\int_{A}^{C}f(x)dx$$

In Equations (2) and (3), *x* represents displacement, and *f*(*x*) represents tensile or compressive force.

According to Equation (1), the larger the *CTI*, the greater the prepreg tack. In the previous studies, only debonding force and debonding energy were considered, without considering the contribution of compression energy during the compression process to prepreg tack. For example, the increase in debonding energy caused by the large compression energy storage during the compression process does not necessarily indicate high tack of the prepreg. Therefore, the introduction of *CTI* to quantitatively characterize and evaluate the prepreg tack can fully consider the two processes of tack formation and tack separation, and can more objectively and accurately characterize the prepreg tack.

#### 2.2.3. Vertical Metal Plate Method

**Figure 3:**
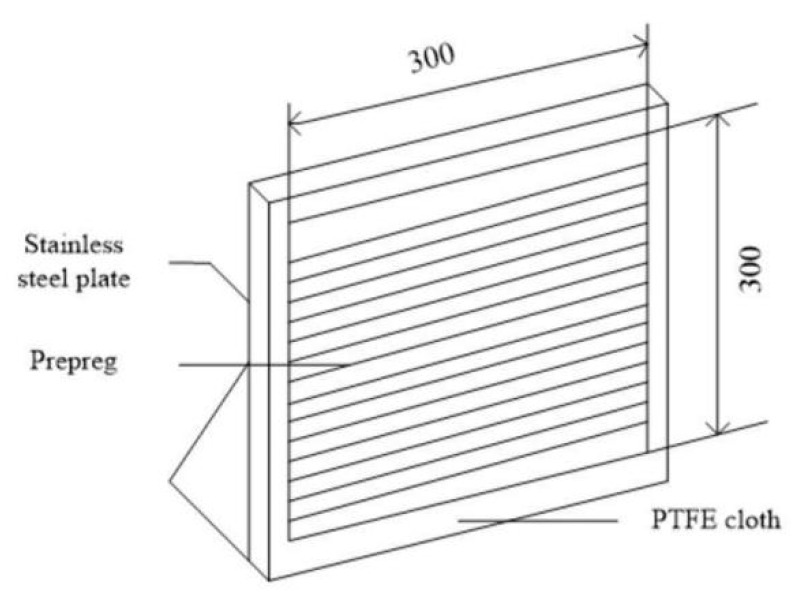
Schematic diagram of vertical metal plate method.

The vertical metal plate method is a qualitative testing method for prepreg tack (Reference standard HB 7736.8-2004 (in China) [3]). As shown in Figure 3, for the prepreg/prepreg tack test, one prepreg specimen with a specification size of 300 mm × 300 mm is pasted to the center of the horizontal stainless steel plate, and the other specimen is laid in the direction of pasting perpendicular to the former specimen; that is, the two specimens are pasted in a 0°/90° direction, with the front and back facing each other. When rubber rollers are used to roll out the air, we can observe the adhesion and non-destructive separation when separating them. The adhesion is observed in the prepreg/stainless steel plate tack test after one hour, according to Figure 3. We conducted the same experiment at least three times. The prepreg tack difference can be divided into five different levels from this test method. The first level has the best laying process for prepregs. When the prepreg tack is at the fourth level, the prepregs themselves cannot adhere to each other and cannot adhere to stainless steel plates, indicating that the prepreg has lost its tack. The tack between the prepreg and the steel plate is observed to separate after one hour of adhering. The five different levels of prepreg tack are shown in Table 2.

## 3. Results and Discussion

### 3.1. Prepreg Tack Test by Compression-to-Tension Method

**Figure 4:**
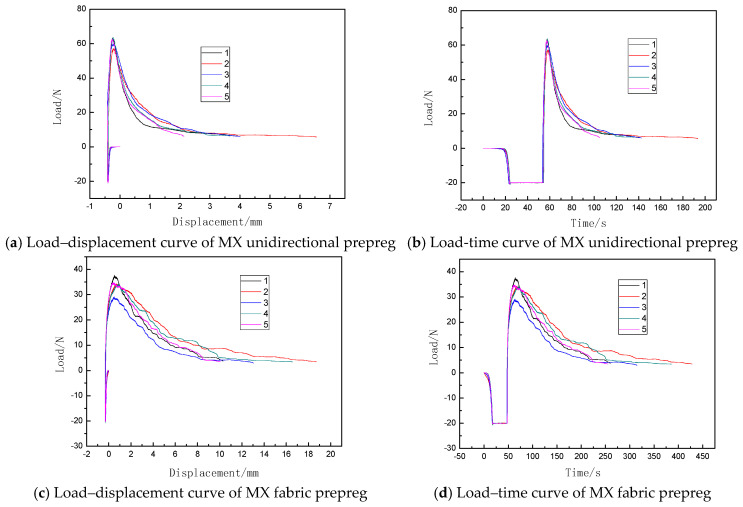
Prepreg/prepreg tack of MX unidirectional prepreg and MX fabric prepreg.

**Figure 5:**
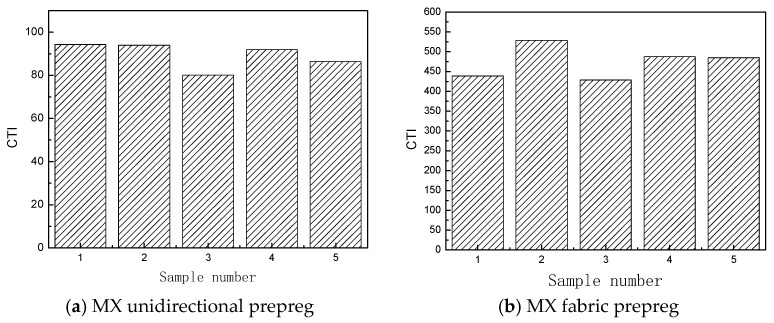
CTI results of prepreg/prepreg tack.

According to the type of reinforcing material, prepregs can be classified into fabric prepregs and unidirectional prepregs. In this paper, the prepreg/prepreg tack of MX unidirectional prepregs and MX fabric prepregs are studied by using the compression-to-tension method, considering the repeatability of multiple tests (Cv) as the evaluation index to carry out the repeatability analysis of the compression-to-tension method. The unidirectional prepregs pasted on the upper and lower fixtures are compressed to tension in a 0°/90° stacking sequence. Figure 4 shows the load–displacement curves of the prepreg/prepreg tack for MX unidirectional prepregs and MX fabric prepregs, while Figure 5 shows the CTI test results of the prepreg/prepreg tack for five different prepreg specimens in the two materials.

From Figure 4 and Figure 5, it can be seen that the results of the prepreg/prepreg tack of the MX unidirectional prepreg test and the MX fabric prepreg test by the compression-to-tension method have good repeatability. The CTI of the MX unidirectional prepreg was between 80 and 94, with a dispersion coefficient of 6.7%. The load–displacement curves of the five specimens almost completely overlapped. At the same time, a higher CTI of between 439 and 529 for the MX fabric prepreg can be observed. The compression-to-tension method is applicable for quantitative testing of the tack of both unidirectional prepregs and fabric prepregs.

By using different characterization parameters, the tack of these two materials will present different results. As shown in Figure 4, the tack of the MX unidirectional prepreg is higher than that of the MX fabric prepreg, using the maximum debonding force to characterize the prepreg tack. However, as shown in Figure 5, the tack of the MX unidirectional prepreg is lower than that of the MX fabric prepreg, using the CTI proposed in this paper to characterize the prepreg tack. But the CTI repeatability of the MX unidirectional prepreg is higher than that of the MX fabric prepreg. Therefore, only considering the tack characterization of the separation process cannot accurately evaluate the tack of different materials. Using CTI to characterize and evaluate the prepreg tack is more accurate than using the maximum debonding force.

**Figure 6:**
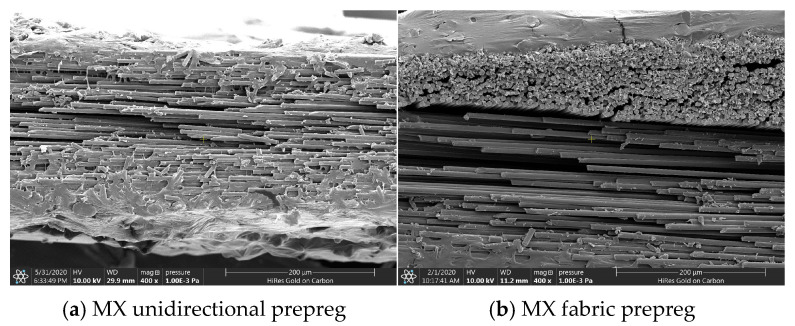
Microtopography of prepreg section.

**Figure 7:**
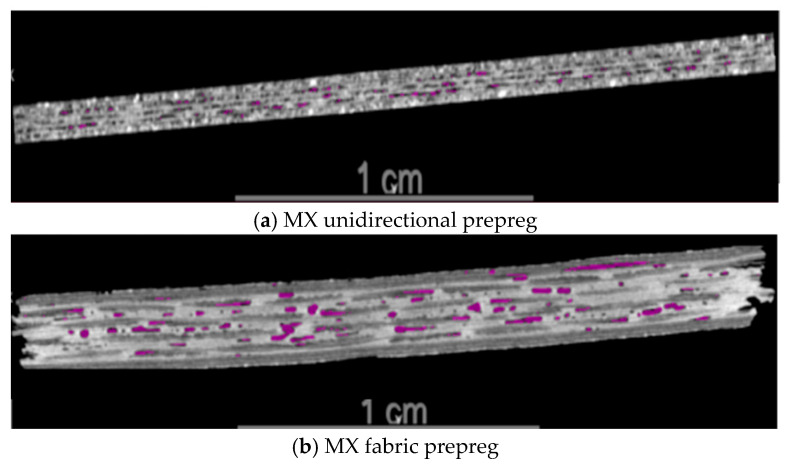
CT scanning micrograph of prepreg stack. (The pink part represents voids).

The tack is closely related to the resin content, morphology, and structural characteristics of these two types of prepregs. The resin content of the MX unidirectional prepreg is 34%, while the resin content of the MX fabric prepreg is 40%. Both types of prepregs are covered with resin film layers on the upper and lower sides. Due to the high resin content, the resin film thickness of the MX fabric prepreg is also higher than that of the MX unidirectional prepreg, as shown in Figure 6. In addition, the initial void content (0.93%) of the MX unidirectional prepreg is much lower than that of the MX fabric prepreg (5.92%), as shown in Figure 7.

As demonstrated by Ahn [20], the prepreg tack is closely related to its four intrinsic material parameters (relaxed modulus, unrelaxed modulus, relaxation time, and initial void content of the prepreg stack). The higher the initial void content, the lower the prepreg tack. The relaxation time is proportional to the resin viscosity characteristics, which depend on the resin viscosity, prepreg fiber/resin content, and prepreg surface properties. The resin content of the MX fabric prepreg is higher than that of the MX unidirectional prepreg, and the relaxation time of the MX fabric prepreg is higher than that of the MX unidirectional prepreg. The void content of the MX fabric prepreg stack is higher than that of the MX unidirectional prepreg. The final test result (Figure 5) shows that the prepreg/prepreg tack of the MX fabric prepreg is higher than that of the MX unidirectional prepreg. It can be seen that the influence of resin content on the prepreg tack is higher than that of the initial void content of prepregs under the same conditions. Increasing the resin content can significantly improve the prepreg tack to a certain extent.

In order to verify the stability of the compression-to-tension method for testing the prepreg tack, three sets of experimental tack data of the MX unidirectional prepreg from three different personnel are shown in Table 3. It can be seen that the average test results of the three different personnel have a good consistency, with below 10% standard deviation. This indicates that the compression-to-tension testing system has good stability.

### 3.2. Comparison Between Compression-to-Tension Method and Vertical Metal Plate Method

The AFP prepreg is usually cut from the prepreg with one meter width. In this paper, the prepreg with one meter width is named the mother prepreg and the AFP prepreg is named the slitting prepreg. The mother prepreg is covered with PE film on top and release paper on the bottom. The slitting prepreg is only covered with release paper on the bottom. Quantitative testing of the slitting prepreg tack has important guiding significance for the setting of the AFP process parameters. This paper applies the vertical metal plate method to qualitatively test the tack of the AX mother prepreg and slitting prepreg, and applies the compression-to-tension method to quantitatively test the prepreg tack. At the same time, this paper compares and analyzes the tack between these two sides (the film surface and the paper surface) and the metal surface.

#### 3.2.1. Vertical Metal Plate Method

**Figure 8:**
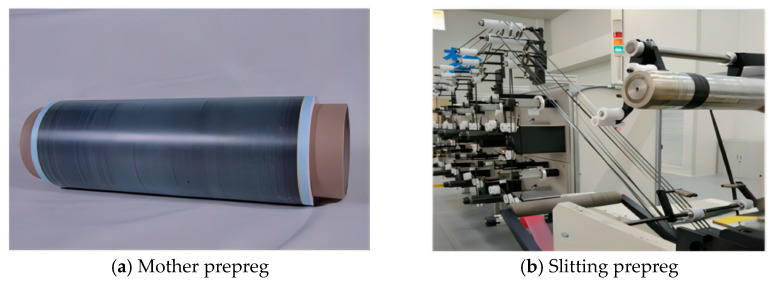
Image of mother prepreg and slitting prepreg.

Table 4 shows the tack test results of the different test surfaces of the AX unidirectional prepreg by using the vertical metal plate method. The images of the mother prepreg and slitting materials are shown in Figure 8. From Table 4, we can see that the tack of the slitting prepreg without PE film is at the fifth level; that is, the prepregs themselves can adhere to each other, cannot be separated without damage, and cannot adhere to stainless steel plates, according to the classification standard in Table 2 [3]. The tack of the paper surface of the prepreg is at the second level; that is, the prepregs themselves can adhere to each other, cannot be separated without damage, and can adhere to stainless steel plates [3]. The test results show that the tack of the paper surface of the slitting prepreg is higher than that of the surface without PE film. The tack of the mother prepreg, whether it is on the film surface or the paper surface, shows a second level, and there is also no significant difference between different batches of prepregs. Due to the fact that the two types of prepregs are adhered with a film (or no-film) surface and a paper surface, the tack of the paper surface is relatively high, resulting in both adhering to each other and not being able to separate without damage. However, the prepreg tack at the second level shows no significant difference.

#### 3.2.2. Compression-to-Tension Method

**Figure 9:**
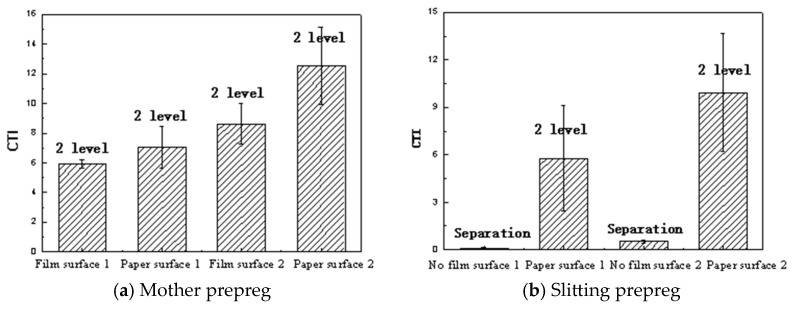
Tack results of compression-to-tension method.

It can be seen from Figure 9 that the compression-to-tension method has a significant differentiation in prepreg tack, which can distinguish the tack of the paper and film surfaces of the prepreg, as well as the differences in the tack between different batches. However, the vertical metal plate method only shows a separation from the stainless steel plates when the CTI is low, and in other cases, it is at the second level. The differentiation of the vertical metal plate method is significantly lower than that of the compression-to-tension method. The PE film without a covering on the surface of the slitting prepreg has a significant effect on the prepreg tack. The CTI of the slitting prepreg without PE film is much lower than that of the mother prepreg with PE film. Almost an order of magnitude difference in CTI can be seen in both the prepregs. Therefore, in order to maintain the prepreg tack, it is necessary to cover PE film on the slitting prepreg or consider other measures to improve its tack. The tack of the mother prepreg cannot represent the tack of the slitting prepreg. The role of the PE film in controlling CTI values for the slitting prepreg is very important to guide the setting of AFP process parameters.

## 4. Conclusions

The compression-to-tension method established in this paper is less affected by the testing personnel and is suitable for quantitative testing of the tack of the unidirectional prepreg and the fabric prepreg. The Compression Tack Index (CTI) is an accurate parameter that characterizes the prepreg tack because it can reflect the process of tack formation and separation. The compression-to-tension method can be used to evaluate the tack level between different materials and evaluate the tack life of prepregs, which has important guiding significance for the AFP process.

The quantitative testing of prepreg tack using the compression-to-tension method has a higher differentiation. The vertical metal plate method only reflects a “fifth level” when the prepreg tack is low, which is significantly lower in differentiation than that of the quantitative testing by the compression-to-tension method.

The tack of the paper surface of the unidirectional prepreg is significantly higher than that of the film surface. The tack of the surface without PE film of the slitting prepreg is much lower than that of the mother prepreg with PE film.

## Figures and Tables

**Table 1:** Performance parameters of prepregs.

Prepreg Type	Resin Content/wt%	Theoretical Calculated Fiber Volume/%	Area Density/g/m^2^	Theoretical Single-Layer Thickness/mm
MX unidirectional prepreg	34	59.2	294	0.184
AX unidirectional prepreg	35	58	223	0.14
MX fabric prepreg	40	55.8	475	0.285

**Table 2:** Five levels of prepreg tack.

Level	Phenomenon
1	Prepregs adhered to each other, separated without damage, and adhered to stainless steel plates
2	Prepregs adhered to each other, separated with damage, and adhered to stainless steel plates
3	Prepregs adhered to each other, separated without damage, and did not adhere to stainless steel plates
4	Prepregs did not adhere to each other, separated without damage, and did not adhere to stainless steel plates
5	Prepregs adhered to each other, separated with damage, and did not adhere to stainless steel plates

**Table 3:** Test results of different personnel.

Person A		Person B		Person C	
Sample Number	CTI	Sample Number	CTI	Sample Number	CTI
1	72	1	51.8	1	54.1
2	69.3	2	52.9	2	55
3	58.5	3	57.9	3	53
4	60.8	4	57.1	4	59.1
5	65.4	5	62.1	5	62.8
6	63.7	6	57.1	6	63.2
7	68.3	7	77.7	7	66.2
8	71.1	8	62.1	8	53.4
9	62.4	9	70.7	9	59.6
10	66.5	10	69.4	10	67.3
Average value	65.8	Standard deviation	61.88	Standard deviation	59.37
Standard deviation	4.47	Standard deviation	8.36	Standard deviation	5.37

**Table 4:** Data sheet of vertical metal plate method for mother and slitting prepregs.

Prepreg Type	Batch Number	Surface	Tack Level
AX unidirectional prepreg—slitting	1	Surface without PE film	5
		Paper surface	2
	2	Surface without PE film	5
		Paper surface	2
AX unidirectional prepreg—mother	1	Film surface	2
		Paper surface	2
	2	Film surface	2
		Paper surface	2

## Data Availability

The original contributions presented in this study are included in the article. Further inquiries can be directed to the corresponding author.
